# Topics of the Month

**Published:** 1893-01

**Authors:** 


### TOPICS OF THE MONTH.

The Pan-American Medical Congress, which will be held in Washington, September 5, 6, 7, and 8, 1893, embraces in its organization the names of the following Buffalo physicians :

Dr. John Cronyn, member of the National Committee on Organization ; Dr. A. A. Hubbell, member of the Auxiliary Committee ; Dr. Matthew D. Mann, Honorary President Section on Obstetrics ; Dr. Henry R. Hopkins, Honorary President Section on Hygiene, Climatology, and Demography ; Dr. Charles Cary, Honorary President Section on Therapeutics ; Dr. Roswell Park, Honorary President Section on Orthopedic Surgery ; Dr. William H. Heath, Assistant Secretary-General ; Dr. John Parmenter, Honorary President Section on Medical Pedagogics ; and Dr. William Warren Potter, Executive President Section on Gynecology and Abdominal Surgery.

We called attention to the fact, in the last number of the Journal, that it is important, in view of the large expense incurred in organizing the Congress, for every one who proposes to become a member, or who desires to receive the benefit of its publications, to register promptly, by sending the fee of $10 to Dr. A. M. Owen, Treasurer, Evansville, Ind. A further important question is that of securing an appropriation from Congress to defray the expenses of entertaining the visitors and to meet the necessary expenses incidental to the sessions. It is essential that members of the Senate and House of Representatives be made familiar with the objects and purposes of this Medical Congress, and that their intelligent support of its purposes be secured. A personal appeal to each member by his medical friends would go far towards accomplishing the desired end.

---

The Buffalo Department of Health has issued a circular, calling attention to the numerous instances of failure to comply with the laws relating to the registration of births. This must be done within three days by every physician and midwife who attends a case of labor. A neglect of this important duty impairs the usefulness of the Records of Vital Statistics. Hence, the Health Commissioner calls upon every physician for a report of all births of which he has been officially cognizant during the year 1892, that have not been heretofore reported.

---

DR. ERNEST WENDE, Health Commissioner, has issued a circular-letter to physicians relating to the exclusion of contagious diseases from the various schools, in which he says, that in reporting cases of contagious diseases among children, every physician "*must state additionally the number of children in the household and the particular school or schools they may attend.*" It is incumbent upon every physician to make the laws efficient by giving cheerful aid to the Department of Health, and we presume this order of the Health Commissioner will be faithfully and literally obeyed.

---

THE American Pharmaceutical Association, through its committee on membership, is anxious to present at a meeting to be held in Chicago, in August, 1893, a long list of names of reputable pharmacists in the United States and Canada. In order to accomplish this purpose, blank applications and full information regarding fees and the benefits of becoming a member of this association, will be furnished by Dr. H. M. Whelpley, 2343 Albion place, St. Louis, Mo., who is the Chairman of Committee on Membership. We hope the pharmacists residing in Buffalo and vicinity will avail themselves of this privilege. Nothing is more conducive to improve a methods than membership in the several local, state, and national societies, and we hope the *esprit de corps* of the pharmacists of Buffalo will be kept at a high standard.

---

THE *United States Investor* announces that it will begin on Saturday, January 7, 1893, the publication of its prize essays upon American Cities and Towns. It will be remembered that in a recent number of the JOURNAL we called attention to the fact that the *Investor* offers $1,000 in prizes for essays not more than one column each, one column to consist, approximately, of 1,000 words. The towns and cities from which essays have already been received number 120, and in the list we notice that Buffalo is represented. These essays will be received until December 31, 1892, when they will be placed in the hands of the judges, who will render their decisions and award the prizes to the successful competitors. All essays that are intended for competition should be marked as such and forwarded to either of the offices of the *United States Investor*, 185 Franklin street, corner Pearl, Boston, Mass.; 335 Broadway, New York ; or 241 Chestnut street, Philadelphia, Pa.

---

THE Southern Pines Resort Company, of North Carolina, announces that the Sand Hills, which is the highest place in the long-leaf pine region, is a most important health resort for those suffering with throat and lung troubles. A number of Northern physicians have been investigating, and recommend it in the highest terms, and several who stand high in the profession have built fine residences at Southern Pines. The place is rapidly increasing in population, and is recorded as the most beautiful location on the line of the Raleigh & Augusta railroad. It now contains about seventy-five fine houses, among which are four hotels, and there is a demand for more cottages. These can be rapidly erected at a very small cost, on account of the cheapness of lumber and labor. For further information, address Hon. John T. Patrick, resident manager, Southern Pines, N. C.

---

THE question of good roads is always an interesting one to physicians, and any considerate move or well-arranged plan looking towards the improvement of highways in the State of New York will receive their cordial support. Col. Albert A. Pope, of Boston, is an enthusiast on this subject, and is also a man of practical ideas relating to it. His proposition is to petition the two houses of Congress to found, in Washington, a Road Department, similar to the Agricultural Department, for the purpose of promoting knowledge in the establishment of or constructing and maintaining roads, with the further suggestion that provision be made in it for teaching students, so that they may become skilled road engineers. It is also proposed that there be established a permanent exhibit, in which shall be shown sections of roads illustrating various methods of construction, and also the best road materials, and machinery. We feel sure that every physician who receives a copy of this petition will gladly sign it, and we hope that it will prove of much service in the direction named. Copies of the petition will be sent to any person interested in the subject by Col. Pope, whose address is P. O. Box "B," Boston, Mass.

The State Commission in Lunacy has issued a circular, under date of December 10, 1892, calling attention to the importance of the clinical teaching of insanity in public hospitals for the insane. The circular quotes the resolutions adopted by the Association of Medical Superintendents in American Institutions for the Insane, at its annual meeting, held at Toronto, Canada, in 1871, that, in substance, advise the teaching of insanity in the medical schools by the ordinary plan of didactic lectures, and that this didactic teaching should be supported by clinical instruction, whenever practicable. There is no doubt, as the circular states, that citizens of the State, of all classes, cannot fail to derive benefit from the diffusion of a more practical knowledge of insanity among the medical profession. We are pleased to note this commendable attempt on the part of the State Lunacy Commission to improve the methods of teaching questions relating to this singular and important disease. The large number of patients in the public hospitals for the insane, suited to the clinical teaching of insanity, ought to render it a comparatively easy matter to carry out the main purposes of the Commission in issuing this circular. This will apply to medical colleges located in large centers, and especially where there are State hospitals for the insane, but we do not see how individual physicians, who may desire to avail themselves of the privilege, can expect to derive much benefit from "such facilities for the clinical study of mental diseases as in the judgment of the medical superintendent may be deemed wise and proper."

